# Determining the Nitrogen Content in (Oxy)Nitride Materials

**DOI:** 10.3390/ma11081331

**Published:** 2018-08-01

**Authors:** Franck Tessier

**Affiliations:** Univ. Rennes, CNRS, ISCR (Institut des Sciences Chimiques de Rennes)—UMR 6226, F-35000 Rennes, France; Franck.Tessier@univ-rennes1.fr

**Keywords:** nitrogen analysis, (oxy)nitride, powder, thin films

## Abstract

Nitrogen (and also oxygen) determination has become an important parameter to characterize (oxy)nitride materials for many properties and applications. Analyzing such anions with accuracy is still a challenge for some materials. However, to date, a large panel of methodologies is available to answer this issue with relevant results, even for thin films. Carrier gas hot extraction techniques and electron probe microanalysis with wavelength dispersive spectroscopy (EPMA-WDS) look attractive to analyze bulk materials and thin films, respectively. This paper gathers several techniques using chemical and physical routes to access such anionic contents. Limitations and problems are pointed out for both powders and films.

## 1. Introduction

In recent decades nitride-type materials have been increasingly studied for diverse applications, for example, in the domains of energy, optics, and the environment [1,2,3,4]. However, the determination of their anionic content often appears problematic in bulk, or in the particular case of thin films. Generally, a combination of several methods is necessary to help determine the real contents, as some measurements can be over- or underestimated by the technology and material’s limits. Overlapping between element detection, water contamination, adsorbed species, and high specific surface area powders are examples of situations that can seriously affect the nitrogen/oxygen analyses.

This paper reviews some of the techniques to determine the nitrogen content from small (as a dopant in oxides [5,6,7]) to large values (in (oxy)nitrides), as well as oxygen as a co-anion in oxynitride or as an impurity in nitrides. Each method is illustrated with a non-exhaustive list of examples. Nitrogen needs to be measured with accuracy and reproducibility to guarantee the reliability of the chemical formulas and to explain its role in ensuing properties and applications. Basic methods and some more sophisticated equipment are presented to access N/O contents in solid materials—thin layers included.

It is also important to point out the necessity to obtain standards of high quality to calibrate the measurements. However, finding calibration standards with high nitrogen contents (10–20 wt% N) may be quite challenging. To date only a few papers have raised the issue of nitrogen analyses [8,9,10].

## 2. Chemical Methods

Nitrogen can be quantified in bulk samples by a series of direct and indirect methods that are presented below. However, none of them give access to an absolute value of the content, and it is often required to compare each result from one method to another. The characteristics of these methods are gathered in Table 1.

Regarding thin films, the difficulty to collect a few mg of matter in thin layers seriously limits the use of these methods. Physical methods (see Section 3) are more relevant for the study of films.

### 2.1. Thermogravimetric Analysis

A TGA analysis enables to determine indirectly the nitrogen content in measuring the weight variation of a nitrided sample transformed into an oxide by heating under oxygen [8]:

MN_x_ + y/2 O_2_ = MO_y_ + x/2 N_2_

The oxidation of the (oxy)nitride sample involves the substitution of two nitrogen atoms by three oxygen atoms corresponding to a relative increase in the anion mass by 71.34% (in the case of no change in the oxidation state of the element M). For example the oxidation of TiN into TiO_2_ should lead to an expected weight gain of 29.06% and that of CrN into Cr_2_O_3_ to 15.14%. Conversely, when an (oxy)nitride is prepared by reacting an oxide precursor under ammonia, the corresponding weight loss may be used to determine the nitrogen content of the product. High-quality data are obtained only if the starting and final states are pure and well-defined phases. The presence of volatile species and impurities will affect the weight changes. The use of a clean reaction vessel avoids any unwanted impurities under NH_3_ thermal treatment and, therefore, subsequent additional variation of the final mass. The thermogravimetric method can produce reliable results, as in the determination of N, C, O elements in AlN resulting from the reaction of Al_2_O_3_, N_2_, and C [11].

### 2.2. Rietveld Analysis

A quantitative approach of the nitrogen content may be obtained by Rietveld analysis. From the unit cell content it is possible to easily deduce the corresponding chemical formula. For oxynitrides, X-ray diffraction does not allow distinguishing between oxygen and nitrogen atoms because of the very close values of their scattering factors. However, neutron diffraction is a more suitable method because the Fermi lengths of these two elements b(O) = 0.58 × 10^−12^ cm, b(N) = 0.94 × 10^−12^ cm are different enough to detect any structural ordering between them. The refinement of the atomic sites’ occupancies leads to oxygen and nitrogen contents, then to a formula for which a comparison with a direct elemental analysis may be recommended. An illustration is given by the Rietveld refinement performed on a SrTaO_2_N sample with no constraint on the overall composition, in excellent agreement with TGA analysis [12]. However, this approach may be limited by the quality of the diffraction data and the nature of the material itself (impurities, structural disorder, poor crystallinity, etc.).

### 2.3. Kjeldahl Method

Johan Kjeldahl (Copenhagen, Denmark, 1849–1900) was initially studying the nitrogen titration in proteins during malt production in the beer brewing process. His method was extended to a large panel of materials: liquid and solid, organic and inorganic. When nitrogen is under an organic form, the experimental protocol involves three steps: dissolution in acids to obtain mineral nitrogen, distillation, and titration [9,13]. The first step consists in heating and degrading the organic nitrogen into an ammonium salt in the presence of concentrated sulfuric acid and catalysts (CuSO_4_, KSO_4_). Then, sodium hydroxide is added and the solution is distilled to convert ammonium into ammonia. Ammonia is swept along with water vapors and then trapped through a water condenser in the receiving acid solution. A basic illustration of the set-up may be found in [14].

Finally, ammonia is quantitatively determined following a back titration (ammonia is captured by a carefully measured excess of a standardized acid solution) or a direct titration (boric acid captures ammonia gas and forms an ammonium-borate complex that is neutralized by the addition of sulfuric acid).

In the case of inorganic samples, i.e., (oxy)nitrides, the Kjeldahl method may be limited to two steps: distillation and titration. The principle of the chemical analysis of nitrogen is based on the reaction of the nitride ion N^3−^ with a strong base and the formation of ammonia, which is then titrated. As an example, AlN reacts with KOH in water to evolve ammonia, which is then titrated with a 0.1 N H_2_SO_4_ solution in the presence of methyl red indicator dye. This determination works very well and is a textbook case proposed to the students during lab works. It was also possible to determine the N content in BN by this method only if a temperature of 260 °C was reached [15], a temperature sufficiently high enough to decompose this nitride.

Although the Kjeldhal method applies for several nitrogen-based materials ((oxy)nitrides, amines, ammonium salts, etc.), it does not fit for nitrates, nitrites, nitrosyls, or cyanides that need to be reduced first as ammonia. For this purpose, Devarda’s alloy (from the Italian chemist Arturo Devarda (1859–1944)) is a well-known reducing powder composed of 50 wt% aluminum, 45 wt% copper, and 5 wt% zinc. Once dispersed in a NaOH solution, nitrates and nitrites are reduced into ammonia, allowing the nitrogen determination by acid titration.

However, the Kjeldahl method presents several limits to determine the nitrogen content of a large number of more refractory (oxy)nitrides, for example, TiN, Si_3_N_4_, SiAlONs, etc., for which the temperature reached in the alkaline solution is not high enough to attack the sample. The Kjeldahl determination gives, in that case, only the opportunity to measure the quantity of adsorbed entities (such as NH_x_, for example) at the surface.

### 2.4. Grekov Method

The Grekov method, so-called after the stay of Grekov in our laboratory, is an alternative to the Kjeldahl method where the product to analyze reacts at higher temperature with melted potassium hydroxide, according to the set-up presented in [8,16].

**Figure 1:**
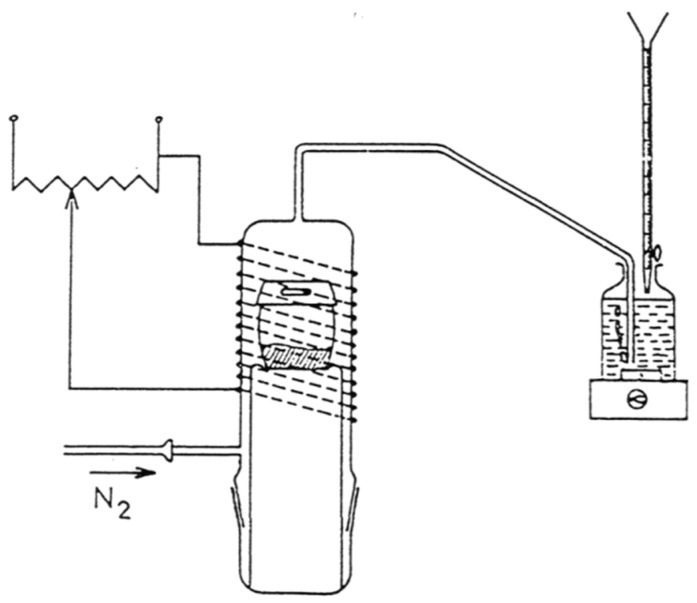
Device used for nitrogen determination.

The sample to measure and KOH pellets are placed in an alumina crucible whose cover is drilled (Figure 1). The reaction takes place in a dry and inert atmosphere at 400–500 °C in a furnace made of a glass tube on which a resistor is coiled. This resistor is embedded in a refractory cement that provides thermal inertia. The released ammonia dissolves in water and is neutralized by a 0.02 N sulfuric acid solution. The method allows the determine of the total amount of nitrogen in the sample with a precision of 1 to 2 wt%. It was applied successfully to nitrided phosphate glasses [17], as well as for nitridophosphates AlPON, GaPON, etc. [18]. However, the attack remains incomplete for transition metal (oxy)nitrides, such as TaON, Ta_3_N_5_, or for the systems Cr-N and Sr-Ta-O-N. The temperature delivered by the electric system is not high enough to totally decompose the samples. An attempt to use the Devarda alloy with tantalum-based nitrides produces no effect to help the nitrogen determination.

### 2.5. Dumas Method

**Figure 2:**
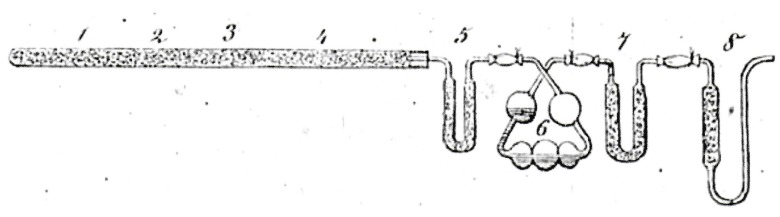
Original set-up developed by Jean-Baptiste Dumas.

The method proposed by Jean-Baptiste Dumas (1800–1884) [19] requires high temperatures to release under oxygen all the nitrogen contained in the sample. The process at the origin of the combustion technique’s devices is briefly described in literature [9,20] (Figure 2).

The sample—weighed and placed in a tin capsule—is heated rapidly under pure oxygen at temperatures between 900 and 1200 °C with oxidizing agents such as V_2_O_5_, CuO, or Cu_2_O. In a first step, the combustion leads to by-products, such as gases (water vapor, CO_2_, O_2_, nitrogen oxides, N_2_) that are then separated and removed using appropriate traps. Nitrogen oxides are reduced into molecular nitrogen through a Cu-based catalyst at 650 °C. N_2_ concentration is determined volumetrically (nitrometer or azotometer) or through a thermal conductivity detector (catharometer).

This method is considered as an alternative of choice to the Kjeldahl determination for faster, easier, and cost-effective use. No toxic or dangerous chemicals/catalysts are needed. The procedure is now commercialized in fully-automated devices under the Dumas nitrogen analyzer.

Meyer et al. reported on the tube furnace oxidation technique with gas chromatographic N_2_ quantification for the determination of nitrogen in refractory metal nitrides [21].

### 2.6. Combustion Analysis

Carrier gas hot extraction (CGHE) techniques are now well developed and represent a suitable solution to get access rapidly to the total nitrogen content. Several companies (Leco, Horiba, Eltra, etc.) market devices with technologies regularly upgraded to overcome the difficulties in accurately measuring N content [22,23]. Oxygen and hydrogen may also be determined simultaneously along with nitrogen on specific analyzers. However, particular attention should be given to the preparation of the samples to remove, as much as possible, the impurities often adsorbed on the surface (CO_2_, OH, O_2_, H_2_O, N_2_, NH_x_, etc.) and, principally, in the case of high specific surface area powders. These analyzers for which accuracy and reproducibility are expected are utilized daily in metallurgy, in the food-processing industry for production control, and also as research tools for chemist or materials scientists to determine chemical formulations as fine as possible.

Purchasing such analyzers has a cost, not only for the equipment itself but also for the required consumables (crucibles, chemicals, catalysts, filters, etc.). Nevertheless, if well calibrated, fine analyses are possible to the ppm level, as this technology was first developed for oxygen and nitrogen inclusions within metals at trace levels. By adjusting the mass of the product to a few mg, tens of percent of oxygen and nitrogen can be determined by relevant detectors. From our experience with Leco^®^ analyzers, the main lines of the analysis are the following [24] and may be transposed to similar equipment: the analyzer uses a self-contained electrode furnace for fusion. A few mg of powder are placed in a tin capsule, which is then introduced into a nickel basket. After outgassing an empty carbon crucible, the as-prepared sample is dropped into it. High current passed through the crucible generates high temperatures close to 3000 °C. The combustion gases flow, driven by a carrier gas (He), is illustrated in a diagram presented in [25].

The oxygen released during the fusion is combined with the carbon of the crucible to form CO and a small amount of CO_2_. CO is converted into CO_2_ through a heated rare earth copper oxide catalyst. Oxygen is measured by infrared detection as CO_2_ in an IR cell. Before measuring nitrogen as N_2_, CO_2_ is trapped by sodium hydroxide coated on a silicate carrier (Lecosorb absorber). Some water is formed during this step and removed with anhydrone (magnesium perchlorate). After removing CO_2_ and H_2_O, a dynamic flow compensator balances the losses by adding carrier gas. Finally the resulting gas contains only nitrogen and can be measured by thermal conductivity in a TC cell.

One key parameter for such analyses is to find relevant calibration standards to perform the calibration with care and precision. Indeed, standards can be purchased from NIST or instrument manufacturers. There is a large choice of compositions for oxygen from ppm to tens of ppm in steel or metals (Cu, Zr, etc.), as well as tens of % in ZrO_2_ and SiO_2_. Concerning nitrogen, the supply is not so abundant. It is easy to find standards with ppm and tens of ppm in steel, but at the percent level Si_3_N_4_ (N, 39.94 wt%) is probably one the rare marketed nitride standards. At lower percentages, ε-TaN (N 7.18 wt%) remains a convenient calibration standard [26]. This phase is prepared by reaction in an autoclave from high-purity tantalum foil under N_2_ pressure at high temperatures (close to 1900 °C) and has the main advantage of presenting a defined nitrogen content. TaON and Si_2_N_2_O may be samples of interest to calibrate both O and N within the same composition, but no certified standards are available. Generally, there is a lack of (oxy)nitride standards. High-quality GaN might also be a calibration standard.

This method remains basically very efficient to rapidly determine the full N content of a sample, in relation with the possible simultaneous measurements of O and H.

## 3. Physical Methods

The following investigation techniques require the use of specific facilities with larger instruments that are appropriate for bulk samples, and even more for the characterization of thin films. A description of these methodologies is provided in [27] and some practical characteristics in Table 2.

### 3.1. Electron Probe Microanalysis

The first electron microprobe was built during the Ph.D. studies of Castaing (1921–1988) under the supervision of Guinier (ONERA, Palaiseau, France—1951). This elemental analysis method known as “microsonde de Castaing”, or electron probe microanalyzer (EPMA), consists of bombarding a sample with electrons (similarly to a scanning electron microscope (SEM)) and to analyze the corresponding X-ray spectrum that is specific to each element. Depending on the spectrometer-type attached to the microscope, two spectroscopies are distinguished: by energy dispersive spectroscopy (EDS), where the detector is a solid state semiconductor converting the energy of each individual X-ray into a voltage signal of proportional size; or by wavelength dispersive spectroscopy (WDS), where X-rays are diffracted and separated over a crystal.

EDS coupled with SEM observations is a semi-quantitative method largely used to determine elemental contents. The calculation of the contents is made by the comparison with a set of data measured on standards under different experimental conditions. For light elements like N and O, the results are characterized by a high level of errors. Moreover, the main peak of nitrogen may overlap with some others as, for example, with the L_1_ peak of titanium. Thus, in LaTiO_2_N, one of those elements will be overestimated or underestimated [28]. For thin films, the weak thickness of the film related to the emission depth of X-rays reveals the contribution of the substrate. This method is not appropriate to obtain absolute contents, but rather relative values, proving first the presence of nitrogen and then allowing the comparison of the contents between several samples [29,30]. EDS helps to determine the cationic ratios, but is not appropriate for light elements [31]. The errors can be reduced only by applying rigorous systematics.

EPMA coupled with WDS brings more reliable results than with EDS systems, due to a higher spectral resolution (superior X-ray peak resolution and greater peak to background ratio, higher sensitivity, etc.). This micro-analysis technique is considered to have the highest reproducibility and accuracy provided the resolution of a few microns is sufficient [9] and the standards are appropriate [32]. As light elements can be analyzed starting from boron, WDS looks suitable to analyze (oxy)nitride thin films. The conclusions of a comparison between EDS and WDS analyses show, for LaTiO_2_N thin films, higher nitrogen contents measured with EDS than WDS, quite similar values for oxygen contents, and lower La and Ti values in EDS mode [28,29].

In the study of LaFeO_x_N_y_ films, WDS measurements have been carried out on thick films allowing the quantification of all elements (La, Fe, O, and N) [33]. Bulk Fe_4_N and pure LaFeO_3_ films were used as standards. To avoid the contribution of the substrate, thicknesses higher than 1 μm were required.

WDS analysis is a suitable method to determine the oxygen and nitrogen contents in oxynitride thin films, and an estimated error of 1 at% is generally acknowledged. However, despite the improved spectral resolution of elemental peaks, some peaks may exhibit significant overlaps that result in analytical challenges (between V and Ti for example). A non-exhaustive selection of articles shows the large panel of WDS applications for transition metal nitrides [34,35,36] or glass fibers [37].

### 3.2. Elastic Recoil Detection Analysis (ERDA)

ERDA is a non-destructive analytical method that allows to precisely determine the nitrogen contents in thin films and the concentration depth profiles without the use of standards. Derived from Rutherford backscattering spectroscopy (RBS), ERDA is based on the elastic recoil of atoms of the material collided with a heavy ion beam of sufficient energy (20–50 MeV). In the case of RBS, the detector is placed behind the sample, whereas in ERDA, the detector is in front. This experimental setup has a cost and requires a source of high energy ions. Many parameters make this technique time consuming and difficult, such as the choice of the incidence angle between the ion beam and sample to collect recoiled atoms, or the perfect combination between source, incidence angle, and the detector. Nevertheless, ERDA provides an accurate quantification of light elements, such as nitrogen, even for low concentrations in the presence of heavy elements. The detection limit is lower than 0.1 at%.

The nitrogen determination previously mentioned for LaFeO_x_N_y_ films [33] was carried out using a Cl^7+^ ion beam of energy 43 MeV produced by a 6 MV HVEE Tandetron accelerator. The recoiled atoms from the sample were detected at a scattering angle of 31° with an energy-sensitive detector able to separate the ion species by pulse shape discrimination, the so-called Bragg ionization chamber. ERDA provides measurements in good agreement with the WDS results, a technique easier to set up in a laboratory. Additionally, some compositions were analyzed using ERDA, as illustrated with the examples of perovskites [10,38] and silicon oxynitrides [39]. Measurement errors for light elements, like O and N, can be estimated to 5, 1, and 0.1 at% for EDS, WDS, and ERDA, respectively [33].

Rutherford backscattering spectroscopy (RBS) consists in bombarding the materials to analyze with an energetic (1–3 MeV) light ion (He^+^) beam in contrast to ERDA. Backscattered ions are then detected with an energy containing all the information about the mass of the collided atom and its corresponding depth in the sample. The depth range is about a micrometer, the depth resolution is around 5 nm, and the detection limit is around 0.1%. Thin films with thicknesses lower than 150 nm are appreciated to allow the deconvolution of the different atomic contributions. An example is given by the study of silicon oxynitride thin film [40].

Nuclear reaction analysis (NRA) is often used in combination with RBS for the fine elemental analysis of light elements from lithium to fluorine. The produced species of this reaction may be a photon or a secondary ion typical of the target nucleus. Quantities of oxygen and nitrogen may be measured by the ^16^O(d,p)^17^O and ^14^N(d,α)^12^C nuclear reactions induced by deuterons of respective energies of 860 and 1450 KeV [41]. The advantage compared to RBS is the possibility to have access to lower concentrations in thin layers. Measurements were performed in the Ti-O-N [42], La-Ti-O-N [41], and SrTaO_2_N [43] systems.

### 3.3. Secondary Ion Mass Spectrometry (SIMS)

Secondary ion mass spectrometry (SIMS) is a technique used to determine the depth concentration profile of films by analyzing ejected secondary ions after ionic bombardment (Cs^+^ for example). The mass/charge ratios of the secondary ions are measured using a mass spectrometer. SIMS has a generally correct sensitivity for most of the elements—including light elements like nitrogen and oxygen—however, only relative information characterizes the resulting compositions. Quantifications may be possible, but with the use of well-defined standards, for which composition and thickness should be perfectly known to return to the absolute determination of the elements in the sample. In most cases SIMS is used to detect the presence of nitrogen and to display the relative evolution of the elements in the function of the thickness [33]. SIMS was used to characterize the nitrogen incorporation in HfO_2_ [44] or perovskites [10,45,46].

### 3.4. Auger Electron Spectroscopy (AES)

Auger electron spectroscopy is another technique of interest to investigate the composition of surfaces and thin films. The principle of this spectroscopy is described in [47]. Compared to XPS, AES requires conductive samples and is more surface sensitive, with a higher spatial resolution of 10 nm (compared to 10 μm for XPS).

The nitrogen incorporation was studied in the ultrathin gate dielectric tantalum oxynitride (TaO_x_N_y_) by AES [48]. Due to the overlapping of the principal nitrogen Auger transition (380 eV) with the Ti (383 eV) one, the nitrogen analysis, therefore, becomes difficult in TiN. A direct nitrogen detection based on the use of a secondary nitrogen Auger transition N KL_1_L_2_ (369 eV) is proposed in reference [49].

### 3.5. X-Ray Photoelectron Spectroscopy (XPS)

The impact of high energy X-rays involves the ionization of the atoms in the material to be analyzed and the subsequent formation of photoelectrons. The measurement of their kinetic energy leads to the determination of their bonding energy in the material. Once the peaks of the spectrum are identified, it is possible to determine the concentration of the elements present in the layer, as the area under the peaks is proportionnal to the number of detected photoelectrons. XPS was successfully applied to self-passivated TaNx surfaces to obtain a direct measurement of the N content [50]. 

## 4. Conclusions

A large panel of techniques is available to determine nitrogen/oxygen contents for most materials. Nevertheless, some caution must be taken in the interpretation of the data regarding the nature and the origin of the N/O species: nitride ion, molecular nitrogen, adsorbed entities (NH_x_, OH, H_2_O, etc.). The synthetic approach may also be a source of error if the precursor is prepared with high specific surface area or using soft chemistry routes for which N/O may be overestimated due to adsorbed species. At the laboratory scale, combustion analyses for bulk and powdered materials and EPMA-WDS for thin films may be considered as analytical methods of choice. Access to larger facilities, when possible, also brings excellent results for the study of thin films, i.e., ERDA, RBS, and NRA analyses.

For most cases, combining several approaches will help to refine the anion amounts and, thus, converge to the real contents.

## Figures and Tables

**Table 1:** Characteristics of the chemical methods.

Techniques	Thin Film	Characteristics
TGA	**×**	- tens of mg - as a first approach - limits due to the presence of impurities and volatile species
Rietveld analysis	**×**	- neutron diffraction for oxynitrides - high-quality diffraction data - suitable for highly crystalline samples
Kjeldahl method	**×**	- acid-base determination - around ten mg - the (oxy)nitrides need to be fully decomposed by KOH around 200 °C in solution
Grekov method	**×**	- alternative to Kjeldahl analysis - acid-base determination - around ten mg - the (oxy)nitrides need to be fully decomposed in melted KOH around 450 °C
Combustion analysis	**×**	- few mg of sample - elemental analyses: N, O, H, C, S - very high temperatures - accurate and reproducible - needs calibration standards - fast, easy method and costly consumables

**×**—the method is not validated for thin films.

**Table 2:** Characteristics of the physical methods.

Techniques	Thin Films	Characteristics
EDS	**×**	- calibration with standards - not appropriate for light elements, allow to determine only their presence, but not accurately - possible overlapping between N and other contributions (i.e., with Ti) - estimated measurement error of 5 at%
WDS	√	- calibration with standards - higher spectral resolution - appropriate from light elements starting from B - thick film (>1 μm) - estimated error of 1 at% - possible overlapping between elements contributions (i.e., between V and Ti) - estimated measurement error of 1 at%
ERDA	√	- non-destructive method, no standards - requires a source of high energy heavy ions - depth profile and elemental composition - accurate quantification of light elements - detection limit <0.1 at% - estimated measurement error of 0.1 at%
RBS	√	- requires a source of energetic light ions - detection limit around 0.1 at% - thin films with thicknesses <150 nm
NRA	√	- complementary to RBS - fine elemental analysis from Li to F - access to low concentrations in thin layers - detection limit 10–1000 ppm
SIMS	√	- depth concentration profile - quantitative interpretation difficult (standards) - sensitive technique to ppb level
AES	√	- requires conductive samples - high spatial resolution - detection limit of 0.1 at%
XPS	√	- depth profile - detection limit of 0.1–1 at%

**×**—the method is not validated for thin films; √—the method is validated for thin films.
